# Obsessive-compulsive symptoms and dissociative experiences: Suggested underlying mechanisms and implications for science and practice

**DOI:** 10.3389/fpsyg.2023.1132800

**Published:** 2023-03-22

**Authors:** Nirit Soffer-Dudek

**Affiliations:** The Consciousness and Psychopathology Laboratory, Department of Psychology, Ben-Gurion University of the Negev, Be’er Sheva, Israel

**Keywords:** obsessive-compulsive disorder, dissociation, absorption, agency, self, embodiment, attention, imagery

## Abstract

A strong and specific link between obsessive-compulsive disorder or symptoms (OCD/S) and a tendency for dissociative experiences (e.g., depersonalization-derealization, absorption and imaginative involvement) cannot be explained by trauma and is poorly understood. The present theoretical formulation proposes five different models conceptualizing the relationship. According to Model 1, dissociative experiences result from OCD/S through inward-focused attention and repetition. According to Model 2, dissociative absorption causally brings about both OCD/S and associated cognitive risk factors, such as thought-action fusion, partly through impoverished sense of agency. The remaining models highlight common underlying causal mechanisms: temporo-parietal abnormalities impairing embodiment and sensory integration (Model 3); sleep alterations causing sleepiness and dreamlike thought or mixed sleep-wake states (Model 4); and a hyperactive, intrusive imagery system with a tendency for pictorial thinking (Model 5). The latter model relates to Maladaptive Daydreaming, a suggested dissociative syndrome with strong ties to the obsessive-compulsive spectrum. These five models point to potential directions for future research, as these theoretical accounts may aid the two fields in interacting with each other, to the benefit of both. Finally, several dissociation-informed paths for further developing clinical intervention in OCD are identified.

## 1. Introduction

In recent years evidence has accumulated to suggest a strong link between obsessive-compulsive disorder (OCD) or obsessive-compulsive symptoms (OCS) on one hand (henceforth OCD/S), and a tendency for dissociative experiences on the other hand, including episodes of depersonalization and derealization (sensations of detachment or estrangement from one’s self or the environment, respectively; American Psychiatric Association [APA], 2013), and “absorption and imaginative involvement” (AII; Carlson and Putnam, 1993), (i.e., a tendency for experiencing immersive attention toward an internal or external stimulus, accompanied by obliviousness to surroundings). Dissociative experiences seem to be a transdiagnostic construct relevant to several psychiatric disorders (Soffer-Dudek, 2014; Ellickson-Larew et al., 2020; Buchnik-Daniely et al., 2021; Lynn et al., 2022). It has been suggested that dissociative experiences may interact in specific ways with various disorders, and those specific interactive paths should be studied to advance the understanding of both dissociation and the respective disorders (Soffer-Dudek, 2014). Nevertheless, as dissociative experiences or symptoms have been mostly related to trauma and trauma-related disorders, the dissociation-OCD/S connection is not intuitive for many researchers and clinicians, most of whom naturally belong to only one of the respective fields (either trauma-dissociation or OCD/S). More importantly, the mechanisms governing this association are poorly understood. The present work is a theoretical formulation, aimed to outline suggested underlying mechanisms, and identify pertinent areas that warrant exploration through future empirical research and may be a focus for personalized interventions for high-dissociation individuals with OCD/S. Although most OCD/S researchers see dissociative episodes as an *effect* of OCD/S, the current formulation suggests that certain dissociative experiences may act as a *cause* as well; specifically, the present work will try to establish that AII may contribute to OCS as well as to the emergence of established cognitive risk factors for OCD.

The sections below will: (1) briefly define common dissociative experiences and discuss their etiology; (2) describe their strong, specific relationship with OCD/S; (3) suggest five different causal models with specific mechanisms that may underlie that relationship; and finally (4) remark on identified areas for future study and possible interventions. Better insight into the processes that generate the link between OCD/S and dissociative processes will advance clinical psychological science within both respective fields, and may also augment existing psychotherapies for OCD, which have proved to be less successful among clients reporting dissociative experiences (Rufer et al., 2006a; Prasko et al., 2009; Semiz et al., 2014).

## 2. Common dissociative experiences

Dissociation is defined in the *Diagnostic and Statistical Manual of Mental Disorders (DSM, 5th ed.)* as “the splitting off of clusters of mental contents from conscious awareness” (American Psychiatric Association [APA], 2013, p. 820). Dissociative disorders, accordingly, “…are characterized by a disruption of and/or discontinuity in the normal integration of consciousness, memory, identity, emotion, perception, body representation, motor control, and behavior” (p. 291). Alongside extreme or rare forms of dissociation (e.g., dissociative identity disorder; DID, in which one experiences oneself as having multiple identities), several types of experiences that are widely considered to be dissociative are quite common. For example, experiences of depersonalization and/or derealization (DEP/DER), are characterized by feeling detached or estranged from oneself, either one’s own mental processes, body, or actions (e.g., individuals may feel as if they were located outside of their body), or from the world (e.g., objects, actions, or events may be perceived as “dreamlike” or as moving in slow-motion). The prevalence of DEP/DER disorder is estimated at 1–2%, but transient DEP/DER sensations are very common, with lifetime estimations ranging between 26 and 74% (Hunter et al., 2004). A recent, integrative theoretical account of dissociative experiences maintains that whereas early, severe, or complex traumatic stress (e.g., childhood physical or sexual abuse) may result in extreme dissociative disorders (DID, dissociative amnesia), mild-to-moderate stress may result in mild-to-moderate dissociative experiences, such as DEP/DER (Buchnik-Daniely et al., 2021; Soffer-Dudek and Somer, 2022). Although DEP/DER may represent symptoms of post-traumatic stress disorder (PTSD; DSM-5, American Psychiatric Association [APA], 2013) or appear as a transient experience during trauma (a phenomenon labeled peritraumatic dissociation, e.g., Marmar et al., 1996), it is also characteristic of panic attacks or overwhelming anxiety that is not necessarily trauma-driven (DSM-5, American Psychiatric Association [APA], 2013; Soffer-Dudek, 2014). Moreover, DEP/DER experiences increased longitudinally following excessive rumination over a span of several months, in a sample of individuals with depression and anxiety but no PTSD, mixed with a community sample (Vannikov-Lugassi et al., 2021). In daily diary studies spanning non-clinical and clinical samples, state DEP/DER symptoms were predicted by daily stress and anxiety (Stiglmayr et al., 2008; Soffer-Dudek, 2017a). DEP/DER experiences also tend to increase following full or partial sleep deprivation (Giesbrecht et al., 2007; Selvi et al., 2015; van Heugten-van der Kloet et al., 2015; Soffer-Dudek et al., 2017) and to improve in accordance with the improvement of sleep (van der Kloet et al., 2012a). Finally, as a stand-alone disorder, DEP/DER disorder is not especially related to childhood trauma (e.g., Michal et al., 2016). To conclude, etiological factors for DEP/DER experiences are broad, ranging from traumatic to non-traumatic stress and from psychological to physiological effects.

An even more common dissociative experience is an empirically derived subscale of the Dissociative Experiences Scale (DES; Carlson and Putnam, 1993), labeled AII, considered by many as a personality tendency representing mild or “non-pathological” dissociation (Butler, 2006). AII represents the disintegration of consciousness in everyday behaviors and contexts; it refers to a tendency to be totally immersed in a stimulus, which often will be one’s imagination or daydreaming, which is especially immersive and vivid (Bregman-Hai et al., 2018), but may also be external such as a movie or a book. It is the narrowing of one’s attention to a fragment of experience, to the point where one is oblivious to surroundings (Soffer-Dudek et al., 2015), at times failing to notice substantial occurrences, or registering them unconsciously (resulting in dissociative amnesia, a compartmentalization or inaccessibility of autobiographical memories). AII could be argued to represent an inclination for often experiencing moments where a person functions in a less integrative way, with a decreased sense of an overarching observer self (Soffer-Dudek and Somer, 2022). Normative dissociation may be manifested, for example, in realizing you are unsure of what you had said to someone, as your mind was “someplace else” during the conversation. Another common dissociative experience is “highway hypnosis,” characterized by driving somewhere yet suddenly realizing that you have no memory for parts of the trip (Williams, 1963). Interestingly, researchers have shown that driving while listening to unrelated auditory content relies on the parallel activation of two separate neural systems, supporting the notion that in certain conditions of normal daily life, our mental functioning is indeed divided (Sasai et al., 2016). Absorbers tend to act on “auto-pilot”; their awareness is focused elsewhere while they are performing routine tasks. This may lead to mistakes (e.g., putting the flour in the freezer instead of in the pantry) or to miscommunication (e.g., answering someone absent-mindedly). Automaticity is a complex concept with many definitions (Moors and De Houwer, 2006); a full review of automaticity is outside the scope of the current work. However, automatic processes are commonly seen as self-regulated actions that run to completion without conscious guidance (Bargh, 1989, 1992, 1997), conscious monitoring (Tzelgov, 1999), or meta-consciousness (Schooler, 2002). The disintegration between awareness or conscious guidance, and performing complex behavior, exemplifies how AII is the common or normative manifestation of the “splitting” of awareness occurring in clinical-level dissociation (Bregman-Hai et al., 2020). Indeed, Butler (2006) has suggested that pathological and “non-pathological” dissociation “…both involve a telescoping of the attentional field to concentrate on a narrow range of experience and the concomitant exclusion of other material (internal or external) from awareness and, to some degree, from accessibility” (p. 45). In line with the idea that AII is characterized by mental disintegration, healthy high AII individuals show decreased neural connectivity (lower EEG coherence) at resting state compared to low AII individuals; The observed decreased coherence in relation to high AII was in the long (frontal-occipital) range, and in the central-parietal short-range pair, both mostly in the left hemisphere (Soffer-Dudek et al., 2019).^1^ This supports the notion of a stable and meaningful personality trait. Indeed, despite the commonness of such experiences, some individuals tend to have them more than others. Below, it will be established that not only are individual differences in AII relevant to psychopathology, but also, that they are relevant specifically to OCD/S. But before, two important caveats on AII and common dissociation in general should be noted.

First, a closely related construct is the similarly labeled trait of “absorption” as measured by the Tellegen absorption scale (TAS; Tellegen and Atkinson, 1974). However, the constructs are not identical: Tellegen’s absorption involves both a narrowing and an expansion of attention (Tellegen, 1982),^2^ whereas AII refers mainly to the narrowing of the attentional span, which relegates the neglected—or dissociated—domains to the periphery of consciousness (Putnam, 1997; Leavitt, 2001). Tellegen’s absorption includes the inclination to experience mystical or enlightening experiences and a sense of elation or oneness with nature, all of which are not included in AII. Indeed, AII and the TAS are only moderately to strongly associated, but do not overlap (*r* = 0.42–0.52; Frischholz et al., 1991; Smyser and Baron, 1993; Levin and Spei, 2004). Moreover, in a factor analysis on *N* = 841 they were virtually discrete; AII was labeled “attentional dissociation” (Carleton et al., 2010). Finally, AII, but not the TAS, involves impairments specific to one’s sense of self and agency (Bregman-Hai et al., 2020), constructs that will be further discussed below.

Second, it is important to mention that structural dissociation theorists do not consider AII or DEP/DER to represent dissociation (e.g., van der Hart et al., 2004). These experiences are viewed by them as alterations in attention and awareness that should not be labeled “dissociative,” as they do not necessarily stem from trauma and do not represent a structural dissociation of the personality into independently functioning agentic parts. Indeed, AII and DEP/DER do not necessarily stem from trauma, and accordingly, have been suggested as transdiagnostic factors relevant for understanding several psychopathologies, including OCD/S (Soffer-Dudek, 2014); however, this does not automatically imply that they should not be considered as mild forms of dissociation. The definition of dissociation does not include etiology but rather describes specific symptoms or phenomena. The discussion of whether they should be labeled dissociative or not is outside the scope of the current work. In line with most of the literature on dissociation, including the DSM-5 (American Psychiatric Association [APA], 2013), The present work will refer to these processes as dissociative. Specifically, DEP/DER disorder appears in the dissociative disorders chapter in the DSM-5 and DEP/DER symptoms have been used in the DSM-5 to define the dissociative subtype of PTSD (American Psychiatric Association [APA], 2013). Thus, the current paper will adhere to this terminology. Regarding AII, it is the subscale carrying most of the variance of the DES (Stockdale et al., 2002; Soffer-Dudek et al., 2015), which is the most widely used measure in the literature aimed to assess dissociation. AII is also very high in the dissociative disorders, including DEP/DER disorder as well as DID (Ross et al., 1995; Leavitt, 1999, 2001; Simeon et al., 2009) and it is associated with a similar neural correlate of decreased coherence as severe dissociation (Soffer-Dudek et al., 2019). Theoretically, an extensive account on why AII is rightfully considered to represent dissociation has recently been published, referring to its properties of decreased agency, incoherent sense of self, increased automaticity, and temporarily compartmentalized behavior and thought, all of which render it a common and mild manifestation of multiplicity, that may represent a diathesis for the development of more extreme dissociative symptoms (Soffer-Dudek and Somer, 2022). The reader is referred to this source for the discussion of whether AII should be considered dissociative.

## 3. The association of dissociative experiences with obsessive-compulsive symptoms

Although AII is usually referred to as “non-pathological dissociation,” several studies have demonstrated its linear association with psychopathology in general (Leavitt, 2001; Levin and Spei, 2004; Armour et al., 2014; Soffer-Dudek et al., 2015; Humpston et al., 2016), and specifically, with OCS (Soffer-Dudek et al., 2015; Soffer-Dudek, 2017a,^2019^; Tapancı et al., 2018; Boysan et al., 2022). More generally, dissociative experiences, as measured with the DES as well as with other scales, have been associated with OCD/S in several studies (for a review see Soffer-Dudek, 2014, although additional studies have been published since). Specifically, clinical-sample studies showed that the presence or severity of several OCS in adults or adolescents diagnosed with OCD were associated with dissociative symptoms (Goff et al., 1992; Grabe et al., 1999; Merckelbach and Wessel, 2000; Lochner et al., 2004; Rufer et al., 2006b; Fontenelle et al., 2007; Belli et al., 2012; Tatlı et al., 2018; Meral et al., 2021). In one study, out of 78 patients diagnosed with OCD, 11 (14.1%) were also diagnosed with a comorbid dissociative disorder, and the general mean score of dissociation symptoms in the sample was close to the clinical cutoff score for suspected dissociative disorders (Belli et al., 2012). Similarly, clinical OCD patients are higher in dissociation scales than healthy controls (e.g., Merckelbach and Wessel, 2000; Tapancı et al., 2018; Tatlı et al., 2018). For example, in one study (Tatlı et al., 2018), controls had a mean DES score of 4.87, whereas OCD patients scored a significantly higher 20.58. In another, it was similarly 8.89 vs. 19.36, also a statistically significant difference (Tapancı et al., 2018). Finally, non-clinical or mixed sample studies repeatedly show that OCS and dissociation are associated (Norton et al., 1990; Watson et al., 2004; Zermatten et al., 2006; Aardema and Wu, 2011; Soffer-Dudek et al., 2015; Soffer-Dudek, 2017a,^2019^; Vannikov-Lugassi and Soffer-Dudek, 2018a; Ellickson-Larew et al., 2020). Moreover, several studies have demonstrated the specificity of the dissociation-OCD/S link, as it could not be accounted for by general neuroticism, psychopathological comorbidities, depression levels, psychotropic medication, attention deficit symptoms, or mind-wandering (Watson et al., 2004; Rufer et al., 2006a; Soffer-Dudek et al., 2015; Soffer-Dudek, 2019). There has even been a measure of “obsessional dissociation” developed, merging the two constructs into a single theoretical dimension (Boysan et al., 2018; Yıldırım and Boysan, 2019).

Obsessive-compulsive disorder is characterized by intrusive, unwanted, repeated, and uncontrollable thoughts, feelings, impulses, or images, and/or repetitive behaviors, usually aimed at reducing the associated anxiety. Some OCD/S researchers have discussed or shown that OCD/S may bring about dissociative and absorptive states due to immersion in obsessions or engaging total attention in compulsive rituals. In other words, they have viewed dissociation as an *outcome* of OCD/S. For example, O’Connor and Aardema (2012) have described how during obsessional thinking, individuals experience the world as if “in a bubble,” in an immersive and dissociative consciousness state. Possibly, the narrowing of attention, which is an inherent element in obsessions and in many compulsions (that need to be performed “just right” or memorized) alters the sense of reality and induces DEP/DER. Indeed, in a very relevant analog study, van den Hout et al. (2009) showed how compulsive-like staring brought about DEP/DER in a non-clinical sample. Specifically, they demonstrated how even relatively short intervals (e.g., 30 s) of concentrated staring at a gas stove (participants were asked not to avert their gaze or blink) paradoxically decreased certainty about perception and increased dissociative experiences. This result joined a series of seminal works by van den Hout et al. (2009) showing that compulsions were not only a result of obsessions/anxiety in OCD but also a driving force or causal factor contributing to the maintenance of the inefficient cycle of uncertainty and checking in the disorder. In this respect, dissociation or the disintegration of experience is viewed as an outcome of compulsive acts.

If indeed dissociation is merely an outcome of OCD/S, it should not necessarily be of significant interest to OCD/S clinicians and researchers. Importantly, however, whereas DEP/DER is indeed likely to represent a consequence or by-product of OCD/S, AII may represent a *risk factor* or augmenting factor in generating or maintaining OCD/S (Soffer-Dudek, 2014, 2019). This proposition is based on the idea that individuals with the tendency to become absorbed, “spaced out,” or dissociated may experience anxiety and confusion following such episodes, which may propel them to perform checking or act out other alleged safety measures. In other words, the narrow attentional spotlight and dissociated/automatic behavior characterizing high AII individuals are not continuously present; occasionally, their awareness returns to an integrated state (Butler, 2006), whereupon they may wonder what just transpired, even regarding their own actions. This uncertainty may cause anxiety, obsessing, and checking in an attempt to substantiate reality (Soffer-Dudek, 2014, 2019). Others have also recently noted that dissociation may be the factor that turns “normal” obsessions into abnormal ones, i.e., into OCD (Boysan et al., 2018), and that dissociative amnesia or lack of confidence in one’s own actions may enhance obsessional doubts (Pozza and Dèttore, 2019). Accordingly, AII demonstrates specificity in predicting OCS, in several ways: (1) AII was the only dissociative factor that predicted a longitudinal increase in OCS (Soffer-Dudek et al., 2015); (2) the relationship of AII and OCS cannot be explained by the association of OCS with attention-deficit symptoms or with mind-wandering (Soffer-Dudek, 2019); and finally, and (3) in a daily diary study, with constructs measured as daily states, time-lag analysis indicated that both AII and DEP/DER were elevated on days in which OCS were elevated; however, only AII proved to also be elevated on the previous day (Soffer-Dudek, 2017a). It is important to note that such a design cannot establish initial causality/etiology, but it can shed light on present-day dynamics. To conclude, OCD/S, DEP/DER, and AII may enhance each other reciprocally, meaning that it is important to better understand the role of dissociation in OCD/S.

This importance is also exemplified in several studies showing that high baseline levels of dissociation in individuals with OCD predict poorer treatment response, or in other words, more treatment-resistant OCD (Rufer et al., 2006a; Prasko et al., 2009; Semiz et al., 2014). In one of these studies, an exploration of subscales revealed that the effect stemmed from AII; Specifically, individuals scoring high on AII at baseline were less successful in their cognitive-behavioral therapy for OCD and were more inclined to drop out, even after controlling for psychopathology and medication use (Rufer et al., 2006a). It has been suggested that the reason that highly dissociative individuals with anxiety disorders are less responsive to treatment is because perhaps they detach from negative emotions that arise during exposure therapy sessions, or because of poor attachment following childhood trauma, which hampers the development of a therapeutic alliance (Spitzer et al., 2007; Semiz et al., 2014). Moreover, traumatic-dissociative flashbacks have been suggested to be indistinguishable at times from obsessional phenomena (Lipinski and Pope, 1994; Semiz et al., 2014). However, detachment following negative emotions could be said about any other disorder as well, and thus does not explain the specificity demonstrated for the OCS-dissociation relationship. In addition, it does not explain the specific relation with AII. As for trauma and flashbacks, although the relation of traumatic stress with dissociation is well-documented, the correlation effect size in most samples is medium (*r* = 0.32 in a meta-analysis; Dalenberg et al., 2012), indicating that the proportion of explained variance is only slightly over 10%, pointing to the need to better understand additional sources of variance in accounting for dissociation. This claim is augmented by the fact that whereas several studies found trauma to be related to OCD/S (Mathews et al., 2008; Lafleur et al., 2011; Berman et al., 2013; Khosravani et al., 2017; Miller and Brock, 2017; Ay and Erbay, 2018), several others did not (Fontenelle et al., 2007; Grabe et al., 2008; Selvi et al., 2012; Visser et al., 2014; Ivarsson et al., 2016). Moreover, in a study exploring both dissociation and trauma as potential predictors of treatment non-response in OCD, only the former was a significant predictor (Semiz et al., 2014). In another study, trauma could not explain the effect of dissociation on OCD (Tatlı et al., 2018). Additional explaining factors that have been mentioned regarding dissociation predicting treatment non-response in OCD are high levels of affect during exposure, inhibiting reality testing, regulation of emotional arousal, and habituation (Rufer et al., 2006a; Semiz et al., 2014). It has also been mentioned that the presence of dissociation could be a marker of more severe obsessive–compulsive symptoms (Goff et al., 1992; Semiz et al., 2014). Yet, most of these mechanisms and explanations are non-specific and vague, leaving a gap in our theoretical understanding of dissociation in OCD.

Additional mechanisms must be responsible for the OCD/S-dissociation link, and the idea presented in this paper is that they are specific to the nature of dissociation as an interruption to an integrated sense of self. Indeed, it is being increasingly acknowledged in the OCD literature that one element characterizing OCD is an incoherent, unstable, or ambivalent sense of self (Guidano and Liotti, 1983; Bhar and Kyrios, 2007; Godwin et al., 2020; Aardema et al., 2021; Llorens-Aguilar et al., 2022). For example, a young adult male with OCD stated to the author of this paper that most of the time, he feels as if he is lying, even though he does not intend to lie, thus describing his continuous subjective experience of unfamiliarity and detachment from who he is and his bafflement as to his own identity. This distance from the spontaneous sense of knowing who you are, what you want, or what you mean, is closely related to dissociation and specifically depersonalization experiences, and may be a core feature of OCD/S, as it is closely associated with doubt and uncertainty. Below, specific constructs that may be at play in the OCD/S-dissociation relationship will be highlighted, each denoting a different causal pattern between the respective constructs. Table 1 presents a summary of the specific mechanisms and models suggested in this paper.

**TABLE 1:** Summary of theoretical models presented in this paper, each with a specific suggested mechanism worthy of further study, and a suggested direction of causality between dissociative experiences and obsessive-compulsive symptoms.

Model number	Suggested mechanism	Causal implication	Model name
Model 1	Inward-focused attention	OCD/S → Dissociation	The inward-focused attention to detachment model
Model 2	Sense of agency	Dissociation → OCD/S	The dissociative experiences to cognitions and symptoms model
Model 3	Sensory integration	Common cause	The deficient sense of embodiment model
Model 4	Sleepiness and dreamlike thought	Common cause + Reciprocal effects	The sleepiness to cognitive deficiencies model
Model 5	Hyperactive imagery system, tendency for pictorial thinking	Common cause	The hyperactive and intrusive imagery system model

### 3.1. Model 1: The inward-focused attention to detachment model

**FIGURE 1:**
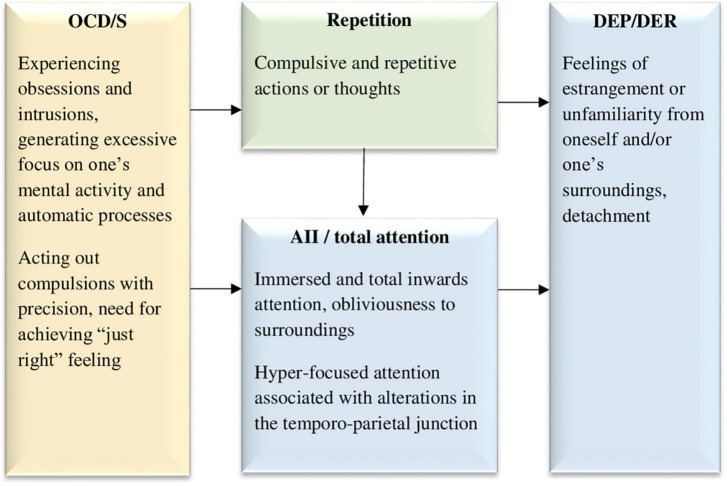
The inward-focused attention to detachment model, hypothesizing that obsessive and compulsive symptoms lead to excessive inward focused attention and repetition, both of which may cause dissociative detachment experiences. OCD/S, obsessive-compulsive disorder or obsessive-compulsive symptoms; AII, absorption and imaginative involvement; DEP/DER, depersonalization/derealization. Yellow boxes represent OCD/S and related phenomena, blue boxes represent dissociative phenomena, and green boxes represent mechanisms involved in their relationship.

AII and DEP/DER, two factors of the DES, are consistently strongly related (e.g., Soffer-Dudek et al., 2015). Moreover, when viewing these factors as states, experience-sampling studies show that AII or daydreaming are accompanied by dissociative detachment (Cardeña and Marcusson-Clavertz, 2016; Vannikov-Lugassi and Soffer-Dudek, 2018a). The hypothesized reason for the strong association between AII and DEP/DER across clinical and non-clinical samples is that *directing attention inward* may bring about DEP/DER (Soffer-Dudek and Somer, 2022). One directional finding for example, is that elevated rumination (entailing inward-focusing), longitudinally predicted an increase in DEP/DER from 1 month to the next in a 6-month study, suggesting a long-term cumulative effect, whereas the opposite effect did not exist (Vannikov-Lugassi et al., 2021). In a different study on people diagnosed with psychosis, self-focused attention and DEP/DER were related, and both associated with voice-hearing engagement (Perona-Garcelán et al., 2020). Another relevant clinical sample is maladaptive daydreamers, i.e., individuals characterized by high AII, who become addicted to immersing themselves in rich, narrative fantasies, at the expense of being present in their realities. Their high DEP/DER levels seem to be related to their sense of “unreal-ness” and detachment when withdrawing from their vivid imagination and returning to what they experience as a dreary reality, engendering sensations of discontinuity in the normal sense of being-in-the-world (Soffer-Dudek and Somer, 2022). Finally, additional indirect support for the notion that excessive inward-attention brings about DEP/DER is evident in the usefulness of grounding interventions to treat DEP/DER, requiring outward-focusing, to reorient the individual to external reality sensory cues (Neziroglu and Donnelly, 2010). Thus, as portrayed in Figure 1, AII or total immersed attention may cause DEP/DER. In OCD/S, intense absorption in ruminations and obsessions engulfing awareness inwardly may lead to dissociative detachment upon exiting the absorbed state (Soffer-Dudek, 2014). As mentioned above, O’Connor and Aardema (2012) have described the obsessive state as an experience of the world as if in a dissociative “bubble.” More recently, in a linguistic-philosophical exploration of the dynamics of OCD, Soffer-Dudek (2023) asserted that OCD clients repeatedly attempt, in vain, to uncover “true” inner states supposedly underlying their own verbal constructions, only leading them to experience a sense of alienation from themselves.

Salkovskis (1998) has noted that individuals with OCD/S tend to allocate their attention to, and monitor, processes that would usually be automatic. Thus, as can be seen in Figure 1, OCD/S may be the beginning of the causal chain, generating DEP/DER through AII. Finally, One of the characteristics of OCD/S that maintains the individual’s attention tirelessly focused inward is *repetition* or perseveration. As mentioned before, van den Hout et al. (2009) have shown how compulsive-like staring brought about DEP/DER in a non-clinical sample. Subsequently, mirror-gazing has been used in several studies as an experimental method of inducing DEP/DER (Caputo, 2010; Brewin et al., 2013; Brewin and Mersaditabari, 2013; Caputo et al., 2021). More generally, different forms of perseveration and repetition may lead to dissociation or breakdown of meaning by blocking the activation of related associations, known as semantic satiation (Pynte, 1991; Sanbonmatsu et al., 2007; van den Hout et al., 2008; Giele et al., 2013, 2014, 2016). In Giele et al. (2016), word repetition was more likely to engender dissociative experiences among individuals with OCD than among healthy participants. The repetitive or compulsive element of these experiences seems to impair normal (automatic) perception, which may explain why rumination, a form of repetitive thinking, was shown to have a directional effect on DEP/DER (Vannikov-Lugassi et al., 2021). It is reasonable to hypothesize that obsessive rumination would have a similar effect, although empirical research is needed to explore this possibility. Figure 1 portrays this idea, whereby OCD/S symptoms inherently carry with them, and perhaps further bring about, both an inward focus of attention and repetition, both of which, in turn, lead to dissociative detachment in the form of DEP/DER.

This model is strengthened by neuropsychological evidence, according to which, event-related potentials in individuals with OCD, stemming from the temporo-parietal junction, seem to point to hyper-focused attention (Mavrogiorgou et al., 2002), and this exact area has been repeatedly implicated in relation to DEP/DER (Simeon et al., 2000; Mantovani et al., 2011), a point which will be further discussed in Model 3. Moreover, functional imaging studies suggest that people with OCD presented with non-affective tasks tend to over-activate regions associated with self-referential processing, including the precuneus and posterior cingulate cortex (Rasgon et al., 2017). Engaging excessively in self-referential processing aligns with the idea of their attention being turned inward at the expense of attunement to their environment, perhaps interfering with an automatic or spontaneous sense of self.

### 3.2. Model 2: The dissociative experiences to cognitions and symptoms model

**FIGURE 2:**
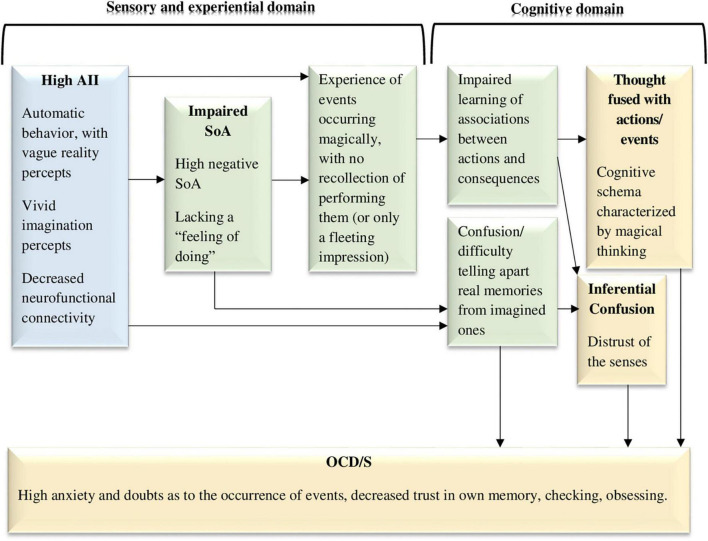
The dissociative experiences to cognitions and symptoms model, hypothesizing that dissociative absorption and imaginative involvement along with impaired sense of agency (negative agency) act as sensory-level and experiential-level vulnerability factors for obsessive-compulsive symptoms and the cognitive schemas that facilitate them (e.g., thought-action fusion). AII, absorption and imaginative involvement; SoA, sense of agency; OCD/S, obsessive-compulsive disorder or obsessive-compulsive symptoms. Yellow boxes represent OCD/S and related phenomena, blue boxes represent dissociative phenomena, and green boxes represent mechanisms involved in their relationship.

It has been previously suggested that AII may play a causal role in enhancing or maintaining OCD/S, especially anxious obsessing and checking compulsions, which are especially related to dissociation (Soffer-Dudek, 2014). Specifically, in the wake of dissociative AII episodes, OCD-related checking may be used to re-orient to the present moment (Soffer-Dudek, 2014, 2019). When ceasing engagement in bouts of AII (e.g., daydreaming, mind-blanking, flow, or other types of immersion), and “waking up” from these moments of decreased self-awareness, individuals may feel a sense of uncertainty regarding their actions in previous moments, and confusion between reality (memories of percepts) and imagination (memories of fantasy). Thus, they may perform checking and repetitive rituals in an attempt to subdue arising anxieties associated with troubling doubts. Indeed, OCD has long been considered a disorder of doubt, and it is well-known that individuals with OCD distrust their cognitive processes (Hermans et al., 2003, 2008; Dar et al., 2022). Thus, as portrayed in Figure 2, confusion between reality and imagination may contribute to OCD/S. Individuals with a natural tendency to often lose their sense of self (i.e., experience many moments of decreased integration–AII), may be more inclined to counteract that feeling and attempt to compensate for it, leading to repeated alternations between moments of decreased and increased awareness to their own thoughts and actions. Thus, a causal influence is hypothesized from AII (in the sensory domain) to the confusion mentioned earlier, which is in the cognitive domain (see Figure 2).

#### 3.2.1. The sense of agency

The understanding of this process may be further enriched by considering the Sense of Agency (SoA), a concept which has been included in the Research Domain Criteria (RDoC) matrix as a promising construct for dimensional research of mental disorders. SoA is the sense that one is causing a physical or mental act (Gallagher, 2000; Synofzik et al., 2008; Morsella et al., 2011), or in other words, the feeling of controlling one’s own actions and, through them, events in the outside world (Beck et al., 2017). Succinctly conceptualized as the sense that “I did that” (Engbert et al., 2008), it is the intuitive impression that our awareness of willing an action to happen is the trigger which initiates the performance of that action. Despite the intuitiveness of this impression, it should not be taken for granted; neuropsychological evidence suggests that brain activity generating an action temporally precedes awareness of initiating the action (Sato, 2009). This means that our idea of causing an action (i.e., SoA) may not actually be the first cue in the causal chain of performing an action, although this is highly unintuitive. SoA seems to arise in tandem (or even after) the advent of action performance. Thus, there are probably biological, psychological, and environmental factors or cues responsible for engendering SoA. It should come as no surprise, then, that there are incidences in which SoA is impaired. Psychopathological impairments in SoA have been investigated and described mainly regarding Schizophrenia (Frith et al., 2000; Gallagher, 2000, 2015; Frith, 2005; Heinks-Maldonado et al., 2007; Synofzik et al., 2010; Voss et al., 2010; Maeda et al., 2013), however, several studies have demonstrated that attenuated or distorted SoA is also a characteristic of OCD/S (Belayachi and Van der Linden, 2009, 2010; Gentsch et al., 2012; Oren et al., 2016, 2019; Giuliani et al., 2017; Fradkin et al., 2019; Szalai, 2019), which will be elaborated in detail below. SoA is often empirically assessed in an indirect (non-self-report) manner through the measurement of intentional binding. Intentional binding is the tendency of most individuals to experience the result of their action as close in time to the advent of that action. In other words, intentional binding is the temporal binding of intentional actions and their effects (Moore and Obhi, 2012), and this represents SoA as it conveys the connection between the action and its consequence.

Although studies combining the concepts of SoA and dissociation are scarce, theoretically, diminished or impaired agency is a core feature of dissociation. In extreme dissociative manifestations such as DID, one experiences significant parts of one’s behavior and mental activity as stemming from a separate agent, located within one’s body. Switching to that state of a seemingly separate agent is often uncontrollable, which is why the disorder has been conceptualized as an impairment in the unity of agency (Kennett and Matthews, 2003). Notably, the degree to which this disorder is mechanistic vs. agentic has been discussed (Cardeña, 1996; Segall, 1996; van der Hart, 1996). Importantly, however, diminished agency is also characteristic of more common dissociative experiences. In AII, one operates automatically, while awareness is focused elsewhere. Thus, behavior and SoA are decoupled, and SoA may be diminished afterward as well, when emerging from the absorbed state (e.g., asking oneself what had just occurred). In DEP, one feels estranged, or detached from, one’s own body, thoughts, or actions (American Psychiatric Association [APA], 2013). This may represent a disruption of both agency and ownership (Sierra and David, 2011; Ataria, 2015a), or the balance between them (Ataria, 2015b) (agency refers to the sense that I am causing an action; ownership is the sense that I am the one undergoing an experience; Gallagher, 2000). In a non-clinical sample, high AII individuals given a free writing task reported feeling less agency during writing, and indeed, were later less successful than low AII individuals at recognizing words extracted from their own text, supporting the notion that their writing process was characterized by decreased awareness (Bregman-Hai et al., 2020). Recent neurofunctional conceptualizations of SoA rely on connectivity between frontal and parietal areas (Haggard, 2017). As mentioned before, reduced connectivity in these areas has been implicated in AII as well as pathological dissociation (Soffer-Dudek et al., 2019; Carbone et al., 2022).

Sense of agency has been studied mainly by experimental manipulation, but recently a self-report questionnaire has been developed, with the aim of capturing individual differences in baseline SoA (Tapal et al., 2017). Some of the items were worded positively (e.g., “I am in full control of what I do”), whereas others were worded negatively (reversed items, e.g., “While I am in action, I feel like I am a remote controlled robot”). Interestingly, the negatively worded items, describing a distortion in the normal SoA, are extremely similar to items from dissociation scales, describing DEP experiences. Positive and negative SoA items were found to load on separate (yet related) factors. This means that experiencing a distortion in normal SoA (e.g., feeling like a robot) is not equivalent to having low levels of normal SoA (e.g., not endorsing the belief that things that one does are subject only to one’s free will), although these two constructs are related and probably causally interact. Possibly, negative SoA (or DEP) is a sensory subjective experience, whereas positive SoA is a cognitive belief (see Synofzik et al., 2013, for a similar conceptualization of two types of SoA); this suggestion, as well as the causal pattern between them, has not yet been established and should be investigated further. Importantly, negative, rather than positive, SoA was related to OCS (Tapal et al., 2017).

Additional recent studies have found impaired SoA in individuals with OCD/S. For example, this was cleverly demonstrated with an indirect linguistic measure; individuals scoring high on OCS tended to omit agency in their spoken language (Oren et al., 2016). These authors also mention that compulsions directly represent deficient SoA, as compulsive action is not experienced as stemming from free will. Deficient SoA in OCD/S has been demonstrated with additional indirect measures, such as diminished suppression of internal expectations in favor of external perception of the consequences of one’s action (Gentsch et al., 2012), impaired ability to identify one’s gaze as bringing about an auditory stimulus (Giuliani et al., 2017, 2021), increased attribution of thoughts as intruders following an auditory cue (Fradkin et al., 2019), and low levels of intentional binding (Oren et al., 2019). According to Szalai (2019), The SoA impairments characterizing OCD are in line with the presence of neurological soft signs (minor, non-localizable deviations in motor or sensory performance) related to motor coordination and sensory integration, compared to controls (Bolton et al., 1999). Thus, as can be seen in Figure 2, dissociative AII may bring about SoA impairments, which in turn contribute to confusion and eventually OCD/S.

#### 3.2.2. Inflated agency

However, it has also been noted that direct (self-report) measures of SoA may produce opposite results, i.e., provide evidence for enhanced SoA in OCD, when those measures assess illusory sense of control (Oren et al., 2016, 2019; Tapal et al., 2017). Indeed, OCD is characterized by a sense of exaggerated control (Reuven-Magril et al., 2008; Oren et al., 2019). This is well-documented in phenomena such as “thought-action fusion” (TAF; Shafran et al., 1996; Shafran and Rachman, 2004) and “thought-event fusion” (Gwilliam et al., 2004), i.e., the notion that thoughts may bring about consequences similarly to actions or events in the world.

How can we conceptualize this apparent contradiction? The apparent discrepancy between indirect and direct measures of SoA may in fact be readily understood when taking dissociation into account. Specifically, if an individual tends to act in an absorbed, automatic state, i.e., tends to miss out on the “feeling of doing” (Belayachi and Van der Linden, 2010) and on the SoA, it may be more difficult for that individual to reliably differentiate actions from thoughts. AII is characterized by a vivid imagination (this was validated this using a mental rotation task; Bregman-Hai et al., 2018), whereas reality is less vivid (because awareness while acting is lacking); thus, absorbers may find it difficult to tell images and memories apart. In other words, considering dissociation may explain why and how individuals come to develop TAF, which is considered as a risk factor for OCD (Shafran and Rachman, 2004). Indeed, Eddy (2016) also suggested that TAF may stem from deficient SoA.

Figure 2 visually portrays this hypothesized dynamic, whereby dissociative experiences, and reduced agency (negative SoA), which are in the subjective sensory-experiential domain, work together in generating both OCD/S and cognitive risk factors for OCD/S. Specifically, acting in the world in an absorbed state, with diminished SoA, may impair the generation of normal associations between actions and their consequences. Moreover, “magical” associations are learned: one repeatedly experiences events as occurring without the person having an accessible representation of performing them. Thus, over time the individual may create a cognitive schema of the world whereby things just seem to “happen,” although one does not explicitly remember, or merely has a fleeting impression of, causing them—an impression which is difficult to tell apart from imagination. Indeed, TAF is related to schizotypy and fantasy proneness (Muris and Merckelbach, 2003).

One may claim that AII may be reminiscent of “inferential confusion”—a reliance on imagination and a distrust of the senses—which is strongly related to OCS (O’Connor and Robillard, 1995). Indeed, dissociation and AII, OCS, and inferential confusion are inter-related (Aardema and Wu, 2011; Paradisis et al., 2015). However, whereas inferential confusion is considered to be a cognitive reasoning tendency to draw inferences on the basis of unlikely possibilities (O’Connor and Aardema, 2012), AII is presumed to be an automatic state, in which attention is narrowed and poorly regulated, without volition or meta-conscious self-awareness (Butler, 2006), perhaps setting the stage for the advent of inferential confusion. Thus, dissociation or AII may be viewed as a rudimentary, experiential construct, that may lead to the development of cognitive schemas which are usually thought of as the source or beginning of the etiological causal chain, including TAF, thought-event fusion, and inferential confusion (see Figure 2).

### 3.3. Model 3: The deficient sense of embodiment model

**FIGURE 3:**
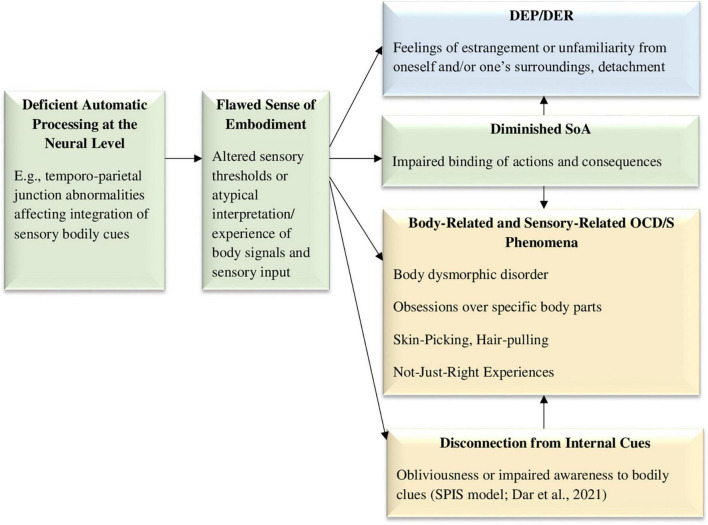
The deficient sense of embodiment model, hypothesizing that a neural mechanism, such as the temporo-parietal junction, is responsible for deficient automatic processing of sensory information, affecting embodiment integration and creating both DEP/DER and body-related OCD/S phenomena. DEP/DER, depersonalization/derealization; SoA, sense of agency; NJREs, not-just-right experiences; OCD/S, obsessive-compulsive disorder or obsessive-compulsive symptoms; SPIS, seeking proxies for internal states. OCD/S and related phenomena, blue boxes represent dissociative phenomena, and green boxes represent mechanisms involved in their relationship.

A prominent theory in the field of OCD/S suggests that individuals with obsessive-compulsive tendencies have attenuated access to identifying their own internal states, known as the “Seeking proxies for internal states” (SPIS) model (Liberman and Dar, 2018; Dar et al., 2021). The lack of connection to one’s own bodily clues may be related to a broader deficiency in automatic sensory processing, impaired SoA, and a flawed sense of embodiment. Indeed, individuals with OCD/S more readily incorporate foreign objects into their sense of bodily self (Jalal and Ramachandran, 2017; Jalal et al., 2020). In recent decades there have been several studies addressing abnormal activity in the temporo-parietal-junction as a neural correlate responsible for bodily sensory integration and the sense of residing within one’s own body (Blanke et al., 2002, 2004; Blanke and Mohr, 2005), and indeed, abnormalities in that area are associated with DEP/DER (Simeon et al., 2000; Mantovani et al., 2011), and have been also detected in OCD (McGuire et al., 1994; Mavrogiorgou et al., 2002; Tan et al., 2013). Moreover, as mentioned before, Mavrogiorgou et al. (2002) found that unlike most psychiatric disorders, in OCD an event-related P300 subcomponent generated in the temporo-parietal-junction may indicate hyper-focused attention. This shared neuroanatomical mechanism implicated in DEP/DER and OCD probably points to deficient multisensory integration (Russo et al., 2014; Eddy, 2016). Thus, a hypothesized impairment in a very rudimentary process of binding together different sensory impressions into an integrated sense of a single embodied self may bring about outcomes related to the mind-body connection, including DEP/DER, and body-related OCD-spectrum symptoms and syndromes such as obsessions over specific body parts, body dysmorphic disorder, excoriation (skin-picking) disorder, and trichotillomania. These phenomena may be associated with altered sensory thresholds or atypical interpretation of body signals and sensory input. As can be seen in Figure 3, temporo-parietal junction abnormalities are hypothesized to generate a flaws sense of embodiment, which in turn may cause DEP/DER, diminished SoA, and body-related OCD/S and related spectrum phenomena.

Mutual mind-body associations also play a part in maladaptive daydreaming, a condition strongly related to both OCD/S and dissociation (Salomon-Small et al., 2021), which often entails repetitive motor stereotypies, as well as very high rates of skin-picking (Somer et al., 2017); these repetitive movements involve the use of the body to enhance focus on one’s internal mental activity, suggesting that further research should be conducted to understand the relationships between attention, internal states, and imagery, on one hand, and bodily cues, on the other.

Another specific OCD-related phenomenon that may stem from deficient automatic sensory processing is the phenomenon of “Not Just Right” Experiences (NJREs), i.e., sensations that things are not as they should be, which is related to OCD/S (Coles et al., 2003; Belloch et al., 2016; Coles and Ravid, 2016). Compulsions may often stem from the need to “make things right” rather than to avoid harm (Ecker and Gönner, 2008; Belloch et al., 2016; Coles and Ravid, 2016). It has been suggested that performing actions with an attenuated SoA and a diminished “feeling of doing” may be the cause for sensing incompleteness and NJREs (Belayachi and Van der Linden, 2010). However, adding dissociation and embodiment into this equation may deepen our understanding of the phenomenon. Specifically, both NJREs and DEP/DER experiences are characterized by a feeling that something is “off,” i.e., not quite as it should be. For example, a NJRE may be knowing that one has washed one’s hands but still not *feeling* that they are clean (van Dis and van den Hout, 2016); similarly, a DEP/DER experience may be knowing that one is staring at one’s own reflection in the mirror, but not *feeling* the expected familiarity. This uncanny impression shared by both experiences may possibly stem from a common neural mechanism, as both DEP/DER and NJREs are experienced in the sensory domain. NJREs have even been conceptualized as “sensory perfectionism” (Coles et al., 2003). Indeed, OCD/S are associated with strong sensory components (Röhlinger et al., 2015; Grimaldi and Stern, 2017) and are characterized by deficient internal motor predictions (Gentsch et al., 2012), suggesting a very basic impairment in the way individuals with OCD/S subjectively experience the world. Thus, DEP/DER and NJREs may stem from a common mechanism, which may be biological, perhaps abnormalities in the temporo-parietal-junction (Eddy, 2016). Figure 3 portrays these hypothesized causalities.

### 3.4. Model 4: The sleepiness to cognitive deficiencies model

**FIGURE 4:**
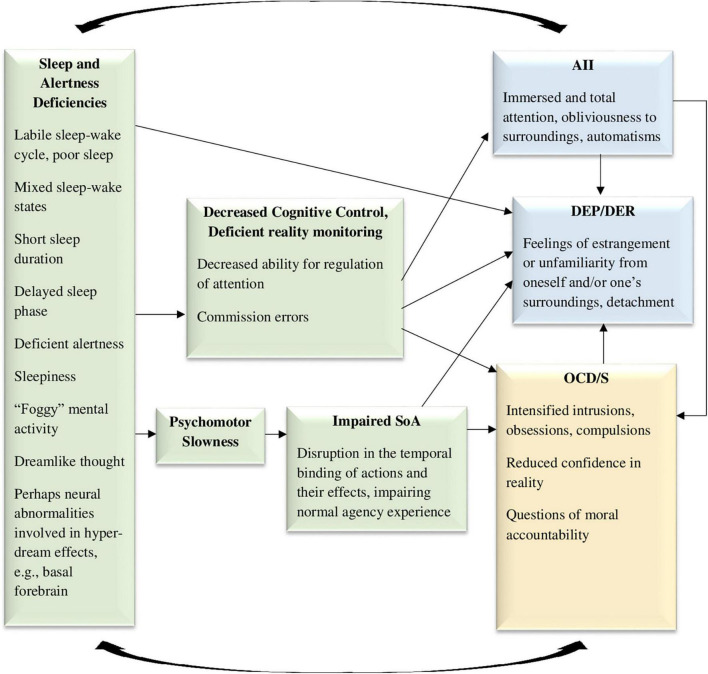
The sleepiness to cognitive deficiencies model, hypothesizing that sleepiness, mixed sleep-wake states, dreamlike thinking, and deficient alertness, may cause both obsessive-compulsive symptoms and dissociative symptoms, through impairments in cognitive control and psychomotor slowness. SoA, sense of agency; AII, absorption and imaginative involvement; DEP/DER, depersonalization/derealization; OCD/S, obsessive-compulsive disorder or obsessive-compulsive symptoms. Yellow boxes represent OCD/S and related phenomena, blue boxes represent dissociative phenomena, and green boxes represent mechanisms involved in their relationship.

In recent years, several studies have demonstrated that sleep impairments are associated with OCD/S, including a large-scale study on a nationally representative sample (Cox and Olatunji, 2016) and several meta-analyses (e.g., Díaz-Román et al., 2015; Nota et al., 2015). Research has shown that OCS are associated with increased sleep-onset latency, reduced sleep duration, reduced sleep efficiency, and delayed sleep phase (Paterson et al., 2013; Boland and Ross, 2015; Nota et al., 2015). It has been claimed that obsessional rumination at bedtime brings about insomnia, similarly to general anxiety and depression (Boland and Ross, 2015), and that comorbid depressive symptoms may be the true cause for sleep disturbance in OCD (Paterson et al., 2013); however, depression and/or anxiety cannot entirely explain the specific relationship between OCD and disturbed sleep (Timpano et al., 2014; Nota et al., 2015; Cox and Olatunji, 2016). It has been demonstrated that in individuals with OCS, impaired inhibitory functions are related to short sleep duration (Nota et al., 2016), suggesting that perhaps a lack of proper sleep makes it harder to inhibit repetitive thoughts and behaviors; this effect was later claimed to stem from deficient alertness, rather than sleep *per se* (Kalanthroff et al., 2017; Naftalovich et al., 2020), and indeed, OCD/S worsens during the times of day that are the most non-optimal according to the person’s chronotype (Naftalovich et al., 2021; and also see Cox and Olatunji, 2022). Thus, as can be seen in Figure 4, it is hypothesized that sleep alterations or deficiencies bring about OCD/S through decreased cognitive control. Interestingly, deficiencies in the systems governing alertness and inhibition are the main suspects implicated in DEP/DER disorder as well (Sierra and Berrios, 1998; Sierra et al., 2002).

More generally, dissociation has also been related to sleep patterns in recent years, specifically, a labile sleep-wake cycle or a tendency for experiencing mixed sleep-wake states (Lynn et al., 2012; van der Kloet et al., 2012b). Dissociation has been conceptualized as sleep elements permeating the waking state (Mahowald and Schenck, 2001; van der Kloet et al., 2012b), and hybrid sleep-wake states are related to automatisms such as sleepwalking episodes, which are considered dissociative (Mahowald and Schenck, 2001). Indeed, sleep and dream patterns characterizing dissociation (discovered by Watson, 2001) may be symmetrically conceptualized as intrusions of wakefulness into sleep (specifically, aroused dreaming patterns or unusual sleep-wake transition phenomena) (Soffer-Dudek and Shahar, 2011; Soffer-Dudek, 2017b). Moreover, dissociative experiences (especially DEP/DER) increased following sleep deprivation in healthy samples (Giesbrecht et al., 2007; Selvi et al., 2015; van Heugten-van der Kloet et al., 2015; Soffer-Dudek et al., 2017), and decreased in parallel to improvements in sleep hygiene in a clinical sample (van der Kloet et al., 2012a). An investigation of four potential mechanisms that may be responsible for the association between rumination and dissociation across two samples, repeatedly revealed the central role of poor sleep quality as the most potent mediator (Vannikov-Lugassi and Soffer-Dudek, 2018b). Thus, as can be seen in Figure 4, sleep abnormalities are also involved in generating DEP/DER. In an opposite causality path, trait dissociation has predictive abilities in the sleep domain; in two healthy high-functioning samples, initial levels of trait AII predicted the extent to which sleepiness increased following sleep deprivation, an effect which remained after recovery sleep as well (Soffer-Dudek et al., 2017). It seemed that high AII individuals experiencing disruption to their sleep cycle had more difficulty in contending with the pull of consciousness toward the sleep state. Thus, AII may represent an impaired ability to regulate, monitor, and control consciousness states, which may be reciprocally related to sleepiness. This is also represented in Figure 4. Notably, sleep deprivation may be one of the conditions in which AII becomes “pathological,” supporting the need for the examination of sleep as an intervening variable when assessing the relation of OCD/S and dissociation.

Interestingly, commission errors, implicated in relation to short sleep in the context of OCD (Nota et al., 2015), are specifically the type of error characterizing dissociators (Giesbrecht et al., 2008). Commission errors in dissociators are probably related to difficulty in differentiating between reality and imagination, as imagination is experienced as vivid and clear (Bregman-Hai et al., 2018). Mixed sleep-wake states probably contribute to this confusion, as reality becomes dream-like. Indeed, dissociation and OCD/S may be related through dysfunctional reality monitoring or reduced confidence in reality (Merckelbach and Wessel, 2000); and this may be brought about by poor sleep.

In a classic, meticulous study of the dream patterns of 361 patients with various neurological impairments or injuries (Solms, 1997), deficits in several anatomical areas were associated with dream reduction or cessation, but certain areas were associated with the opposite: higher frequency of dreaming, higher vividness, dreams experienced as realistic, and dreams pervading waking to the point of confusion between dream and reality. Specifically, these hyper-dreaming effects characterized patients with injuries in the medial prefrontal cortex, anterior cingulate cortex, and basal forebrain (Solms, 1997); accordingly, all those areas have been implicated as neuroanatomical mechanisms involved in OCD as well (e.g., see Sturm et al., 2003; Aouizerate et al., 2004; Modirrousta et al., 2015, respectively). This relates to another possible shared mechanism, namely, excessive imagery, but this will be further discussed in Model 5.

Another sleep-related mechanism which may be responsible for the dissociation-OCD/S relationship may be psychomotor slowness, which has been associated with short sleep duration (Kronholm et al., 2011). Possibly, such slowness may reduce body/brain integrity (discussed in the previous see section “3.3. Model 3: The deficient sense of embodiment model”), and thus may bring about dissociation and sensations of unreality. Indeed, intentional binding or sensory integration is contingent upon short time frames between intention and action (Ismail and Shimada, 2016), and thus psychomotor slowness following short sleep in OCD/S may perhaps diminish SoA and create DEP/DER. ^3^ Such alterations in the sense of reality and agency may undermine certainty in actions and their consequences (as well as memory and attention; Hermans et al., 2008) and thus aggravate OCD/S. In turn, aggravated OCD/S will further disrupt sleep through insomnia, leading to a vicious cycle. Thus, Figure 4 shows psychomotor slowness as a possible mediator between sleep impairments and OCD/S, through impaired agency.

Finally, it should be noted that dissociative sleep-related automatisms (e.g., sleepwalking) are actions done with very little awareness and SoA, which generates questions regarding moral accountability for these acts (e.g., Broughton et al., 1994; Levy and Bayne, 2004). This may be a useful model for understanding the experience of individuals with OCD/S, who act automatically with little awareness and impaired agency, leading them to ask themselves such questions exactly. Indeed, it has been recently suggested that OCD is associated with a distrust of automaticity (van den Hout et al., 2019).

The various paths through which sleep impairments may explain the OCD/S-dissociation relationship are depicted in Figure 4.

### 3.5. Model 5: The hyperactive and intrusive imagery system model

**FIGURE 5:**
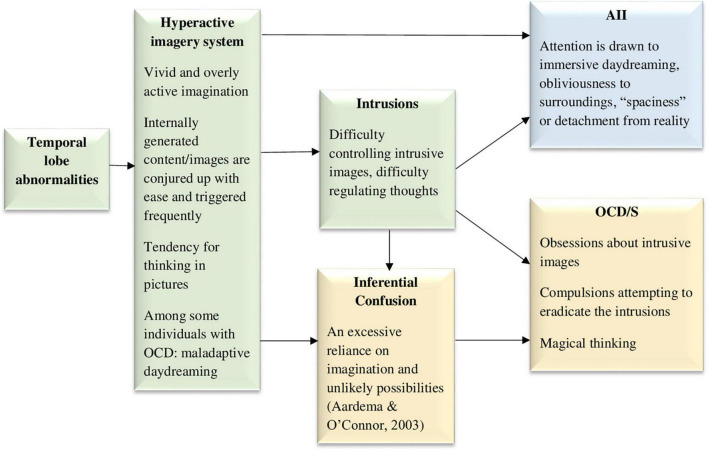
The hyperactive and intrusive imagery system model, hypothesizing that an excessively active and vivid imagery system, causing intrusive images, underlies both dissociative absorption experiences and obsessive-compulsive symptoms. AII, absorption and imaginative involvement; OCD/S, obsessive-compulsive disorder or obsessive-compulsive symptoms. Yellow boxes represent OCD/S and related phenomena, blue boxes represent dissociative phenomena, and green boxes represent mechanisms involved in their relationship.

Intrusive imagery is a central feature in OCD/S (Rachman, 2007; Lipton et al., 2010; Bouvard et al., 2017). In fact, most OCD obsessions seem to involve images (Rachman, 2007; Speckens et al., 2007), either associated with distressing past events (Speckens et al., 2007) or not associated with memories (Lipton et al., 2010). There are individual differences in peoples’ ability to imagine things clearly, vividly, and immersively, and in the frequency of their tendency to do so (Bregman-Hai et al., 2018; Merckelbach et al., 2022; Soffer-Dudek and Somer, 2022). Possibly, an especially vivid imagination in some people means that internally generated content including vivid images are conjured up with ease and triggered frequently, which may leave them at risk for developing OCD/S (see Figure 5). It could be that people who develop OCD/S are those who tend to engage in pictorial or visual thinking above average. Notably, pictorial thinking cannot include negation; thus, a thought such as “I will never hurt my baby” would paradoxically conjure up an image of the person hurting their baby. The salient or vivid nature of the mental imagery may render it harder to get rid of or control, i.e., more intrusive. This may lead to AII, specifically, attention and awareness becoming engulfed in immersive daydreaming, obliviousness to surroundings, or a detached “zoning out.” Hence, Figure 5 portrays a hyperactive imagery system and intrusions as causal factors in both AII and OCD/S. At the cognitive level, a hyperactive and vivid imagery system would also result in imagined possibilities being more easily confused with reality, i.e., inferential confusion; this means that individuals rely excessively on their imagination at the expense of trusting their sensory input, in turn strengthening OCD/S (O’Connor and Robillard, 1995; Aardema and O’Connor, 2003; Aardema et al., 2005). Obsessions may begin as daydreams or imaginings which are then treated as realistic possibilities, further developing into fixations (Aardema and O’Connor, 2003). Indeed, in a general student sample inferential confusion and AII were strongly related, and both were predictive of OCD/S (Aardema and Wu, 2011). Notably, when including both inferential confusion and AII in a model predicting OCD/S severity in a clinical OCD sample, neither were statistically significant (Pozza et al., 2016), probably due to their largely shared predictive ability, which was not included in either construct’s unique contribution estimate. Thus, the figure includes inferential confusion as another possible mediator.

It is noteworthy that there is a clinical condition in which the core problem is excessive imagery, with vivid, intensely emotional, and narrative fantasy activity, namely, maladaptive daydreaming (Somer, 2002). Maladaptive daydreamers compulsively engage in this activity, which they feel a constant urge to perform. Maladaptive daydreaming is different than OCD/S because it is gratifying in the short run, characterized by reward-seeking—similar to a behavioral addiction—rather than necessarily to threat avoidance. Nevertheless, astoundingly high rates of maladaptive daydreamers have either comorbid OCD/S or OCD-related spectrum disorders, such as skin-picking disorder and body dysmorphic disorder (Somer et al., 2017; Salomon-Small et al., 2021). Related to the latter, research has also shown that intrusive imagery is even more salient in OCD with comorbid body dysmorphic disorder than in OCD alone (Nakata et al., 2007). Notably, maladaptive daydreaming is strongly related to AII and has been suggested to represent a dissociative disorder (Soffer-Dudek and Somer, 2022). An investigation of OCD/S in a sample of over 500 maladaptive daydreamers found that approximately 56% of them were over the cutoff for suspected OCD, and dissociation was a strong mediating factor explaining the relationship of OCD/S and maladaptive daydreaming (Salomon-Small et al., 2021). This supports the idea, portrayed visually in Figure 5, that at least for some individuals, a hyperactive imagery system may underlie both OCD/S and dissociative experiences.

Maladaptive daydreamers report higher sense of presence in their daydreams (Bigelsen et al., 2016), suggesting that they may have a low threshold for experiencing percepts and sensations as vivid or real. Anomalies in imagination or an overly active vivid imagination is also associated with magical thinking, paranoid ideation, and even the psychosis spectrum (Rasmussen and Parnas, 2015; Zsila et al., 2019; Merckelbach et al., 2022; Rasmussen et al., 2022). In the neuropsychological realm, the temporal lobes are known to be involved in generating percepts and have been suggested as a mechanism that may relate psychosis and creativity (Flaherty, 2011); perhaps an overly active temporal lobe may stand at the bottom of both excessive imagery and magical thinking. Indeed, the temporal lobes have been associated with paranormal beliefs, anomalous perceptual experiences, and magical thinking (Persinger, 1984; Persinger and Valliant, 1985; Persinger and Makarec, 1987; Bell et al., 2007; Thalbourne and Maltby, 2008). Specifically, the inhibition of left lateral temporal activation successfully suppressed magical thinking, assessed in that study as the tendency to report meaningful information based on random noise (Bell et al., 2007). One study also showed that anomalous experiences (e.g., sensory experiences, sense of presence) can be induced in some people by applying weak magnetic fields from outside the skull in the temporo-parietal area (Persinger and Healey, 2002), implicating the same area already discussed in Model 3 when it comes to unusual embodied sensory experiences. Interestingly, there is evidence of the involvement of temporal lobe abnormalities in OCD, based on consistently higher rates of OCD/S in patients with temporal lobe epilepsy, compared to either idiopathic generalized epilepsy or non-epileptic controls (e.g., Isaacs et al., 2004; Monaco et al., 2005; Ertekin et al., 2009). Temporal lobe epilepsy is also associated with increased vivid nightmares (Silvestri and Bromfield, 2004). Thus, temporal lobe abnormalities are included in Figure 5 as a possible causal factor generating the excessive imagery.

## 4. Discussion

In the present work, five different theoretical causal models were presented, explaining the strong and specific relationship of dissociative experiences and OCD/S, none of which rely on general constructs such as trauma or detaching from negative emotion–constructs that are usually considered as the immediate etiological factors of dissociation, but are not specific to OCD/S. The five models do not negate one another but may be complementary, in other words, may all be true; they are presented separately for the sake of theoretical clarity. However, dissociation and OCD/S may reciprocally affect each other as well as stem from several common mechanisms.

Indeed, although the models were presented as separate, they are closely related. For example, both Models 2 (SoA) and 3 (Embodiment) focus on impaired binding or integration of sensory and/or motor systems; and both Models 4 (Sleepiness) and 5 (Imagery) imply enhanced salience of visual thought during waking. All five models probably work together in contributing to the anatomical-functional-psychological infrastructure generating and/or maintaining OCD/S. For example, attention overly focused inward (Model 1) may reciprocally affect and be affected by, decreased sensory integration (Models 2, 3), and that internal attentional spotlight may be directed toward salient, uncontrolled images (Models 4, 5), which further demand their share of attention, leaving the individual in a vicious cycle of fixation on fantastical possibilities.

More research is needed concerning self-concept clarity, identity, and automaticity in OCD/S. Additionally, AII, SoA, embodiment, sleep-wake boundaries, and imagination activity all seem to be worthwhile avenues for further investigation regarding OCD/S etiological and maintaining factors. In particular, there is a need for experimental work on dissociation and OCD/S, exploring the possibility of dissociative experiences as a causal factor. For example, experiments could explore whether manipulating individuals into an immersed and absorbed state while they perform a task with little awareness may bring about anxiety and uncertainty regarding their automatic actions. Also, experience-sampling and prospective-longitudinal designs are scarce in the OCD/S field; those could shed light on the day-to-day temporal dynamics of the OCD/S–dissociation relationship, as well as the long-term effects of personality. Specifically, large-scale longitudinal developmental studies may reveal whether experiential inclinations such as trait AII may, over time, engender cognitive schema risk factors for OCD/S, such as TAF.

The present work builds upon previous etiological knowledge accumulated on OCD/S, such as inferential confusion, TAF, and the SPIS model. However, considering dissociation adds important insights with incremental value for understanding OCD/S. For example, adding an experiential and sensory perspective helps shed light on the development of cognitive risk factors such as inferential confusion and TAF. Notably, the SPIS model suggests that TAF stems from lack of access to one’s feelings and motivations, and therefore trying to compensate by assigning excessive importance to one’s own thoughts and images (Dar et al., 2021). The current theoretical formulation postulates that considering dissociation is crucial to understanding how it may be that a person may feel diminished access to their own feelings and motivations, and offers a somewhat more specific explanation for TAF, which is characterized by magical thinking. Again, it does not contradict or negate the explanation suggested previously but enriches its nuances.

High-dissociation individuals are known to gain less from ordinary treatment for OCD (Rufer et al., 2006a; Prasko et al., 2009; Semiz et al., 2014), which is why the current formulations may be important in inspiring the development of clinical interventions for such individuals. The models suggested in this work harbor practical clinical significance in several domains. First, future studies could examine whether individuals with OCD may benefit from the use of grounding techniques, usually associated with treating dissociation, to focus their attention outward and ground their sense of bodily self when getting caught up in obsessions or negative emotion. This was briefly mentioned as a useful aid in two independent case reports of clients with OCD (Proescher, 2010; Cheli et al., 2020). Second, the idea that AII may contribute to OCD/S (Models 2 and 4) means that understanding dissociative-absorptive qualities in OCD may impact interventions. For example, one study aimed to enhance attentional abilities in OCD patients by training them at selective attention, but that did not decrease their symptoms (Haug et al., 2013). The present conceptualization, according to which individuals with OCD have a tendency for an absorptive, narrow attentional spotlight, in the form of AII, explains why training them to attend selectively to a single stimulus was unlikely to alleviate their symptoms. In contrast, the training of patients with OCD for attentional control should perhaps focus on the development of the ability to integrate mental states and multitask. Moreover, empathically addressing clients’ sensory experiences of increased automaticity, decreased agency, and resulting reality-fantasy confusion may help them feel better understood by the therapist, which may facilitate their motivation to correct their cognitive oversights.

Third, future studies should explore whether alterations in automatic processing and integration of bodily cues (Model 3), perhaps due to temporo-parietal abnormalities, may also be of importance as a possible focus of intervention. Such a neuropsychological intervention has been attempted in the field of dissociation (Mantovani et al., 2011). Fourth, the idea that sleepiness and mixed sleep-wake states may be involved in the etiology or maintenance of OCD/S (Model 4) means that at the beginning of treatment, an OCD client should be assessed not only with dissociation scales, but also pertaining to sleep quality and sleep-wake boundary phenomena. Sleep-wake boundaries are important to consider, rather than merely focusing on traditional sleep quality measures (Soffer-Dudek, 2017b). For example, a client may off-handedly report that they have no problems falling asleep and indeed, report that they drink coffee every evening and still fall asleep within minutes. This may suggest an inclination for experiencing mixed states, that may manifest in both nighttime aroused dreaming and daytime dissociative experiences, that may possibly exacerbate their OCD symptoms. While this suggestion is speculative and in need of further study, it should be considered as it is quite easy to implement sleep hygiene interventions in addition to treatment as usual. Finally, a hyperactive imagery system (Model 5) may suggest the existence of comorbid maladaptive daydreaming. If the client experiences elaborate, narrative, absorbing fantasies, which they engage in compulsively, treatment should address that, for example by monitoring the imagination activity and its triggers (Herscu et al., 2023). Maladaptive daydreaming often involves an idealized version of the self (Somer, 2002) and experimenting with different personalities (Soffer-Dudek and Somer, 2022), meaning that addressing self-image, self-coherency, or other underlying self-related issues may also be relevant.

The present work has several limitations. Specifically, knowledge of the neuropsychological underpinnings of dissociative experiences is quite scant and preliminary, which makes it difficult to identify the functional source of dissociative impairments in OCD/S. Second, the definitions of dissociative experiences may be at times quite hazy and controversial (e.g., see van der Hart et al., 2004), with points of overlap with other existing constructs such as negative SoA, for DEP/DER, and deficits in attention, for AII (e.g., see Carleton et al., 2010, who defined AII as “attentional dissociation”). Importantly, though, attention deficit and mind-wandering have been shown to be separate factors from AII, and the latter had significant incremental value over them in predicting OCD/S (Soffer-Dudek, 2019). Still, overlapping or ambiguous definitions hinder scientific clarity. Finally, a lack of specific intervention protocols for mild dissociative states limits the ability to tailor treatments differently for individuals with OCD who are high in dissociation. Nevertheless, the use of grounding techniques and the implementation of sleep hygiene seem to be promising avenues.

Hopefully, the present work may encourage further empirical research into the dissociative aspects of OCD/S, as well as establish dissociation as a transdiagnostic factor relevant to additional psychopathologies rather than trauma alone. These novelties carry theoretical significance for the understanding of a link between clinical symptoms which is currently poorly understood and under-researched. Such explorations will enrich both respective fields.

## Data availability statement

The original contributions presented in this study are included in the article/supplementary material, further inquiries can be directed to the corresponding author.

## Author contributions

NS-D was solely responsible for the literature review, conceptualization, writing, reviewing and editing, and any other work associated with this manuscript.

## Footnotes

1 Decreased coherence or connectivity also seems to be a neural marker of dissociation in general, either in healthy participants (Carbone et al., 2022), or in clinical samples including PTSD (Tursich et al., 2015) and DID or dissociative disorders (Hopper et al., 2002; Farina et al., 2014). It is also an established correlate of hypnosis (van der Kruijs et al., 2014), a state which is conceptually close to AII.

2 Tellegen (1982). Content categories: Absorption items (revised). University of Minnesota, Unpublished manuscript.

3 Note that slowness following short sleep is probably independent of the phenomenon of “obsessional slowness” (Rachman, 1974; Veale, 1993), although obsessional slowness might also be hypothesized to bring about diminished SOA and dissociation.
